# Herbal melanin inhibits colorectal cancer cell proliferation by altering redox balance, inducing apoptosis, and modulating MAPK signaling

**DOI:** 10.1186/s12935-020-01206-x

**Published:** 2020-04-16

**Authors:** Omar Al-Obeed, Adila Salih El-Obeid, Sabine Matou-Nasri, Mansoor-Ali Vaali-Mohammed, Yazeid AlHaidan, Mohammed Elwatidy, Hamad Al Dosary, Zeyad Alehaideb, Khayal Alkhayal, Adil Haseeb, James McKerrow, Rehan Ahmad, Maha-Hamadien Abdulla

**Affiliations:** 1 Colorectal Research Chair, Department of Surgery, King Khalid University Hospital and College of Medicine, King Saud University, PO Box 7805 (37), Riyadh, 11472 Saudi Arabia; 2 Department of Biobank, King Abdullah International Medical Research Center, King Saud Bin Abdulaziz University for Health Sciences, Ministry of National Guard Health Affairs, PO Box 22490, Riyadh, 11426 Saudi Arabia; 3 Faculty of Pharmacology, Ahfad University for Women, Khartoum, Sudan; 4 Cell and Gene Therapy Group, Medical Genomics Research Department, King Abdullah International Medical Research Center, King Saud Bin Abdulaziz University for Health Sciences, Ministry of National Guard Health Affairs, PO Box 22490, Riyadh, 11426 Saudi Arabia; 5 Department of Physics, Faculty of Science, University of Khartoum, Khartoum, Sudan; 6 Skaggs School of Pharmacy and Pharmaceutical Chemistry, University of California, La Jolla, San Diego, CA USA

**Keywords:** Herbal melanin, Colorectal cancer, Proliferation, Apoptosis, Reactive oxygen species, TLR4 receptor, Signaling pathways

## Abstract

**Background:**

Colorectal carcinoma is one of the most deadly cancers that requests effective and safe chemotherapy. Evaluation of natural product-based anticancer drugs as adjuvant treatment with fewer side effects is largely unexplored research fields. Herbal melanin (HM) is an extract of the seed coats of *Nigella sativa* that modulates an inflammatory response through toll-like receptor 4 (TLR4). This TLR4 receptor is also involved in the modulation of apoptosis. We therefore explored the anticancer potential of HM and specifically its effect on the molecular mechanisms underlying adenocarcinoma and metastatic colorectal cancer (mCRC) cell death in vitro.

**Methods:**

Cell viability was evaluated using the MTT assay. Cellular reactive oxygen species (ROS), glutathione levels, and apoptotic status were assessed using fluorometric and colorimetric detection methods. HM-induced apoptotic and other signaling pathways were investigated using Western blot technology and mitochondrial transition pore assay kit. TLR4 receptor downregulation and blockade were performed using siRNA technology and neutralizing antibody, respectively.

**Results:**

Our results showed that HM inhibited the proliferation of the colorectal adenocarcinoma HT29 and mCRC SW620 cell lines. Furthermore, HM enhanced ROS production and decreased glutathione levels. HM-induced apoptosis was associated with mitochondrial outer membrane permeability and cytochrome c release, inhibition of the Bcl2 family proteins, and activation of caspase-3/-7. In addition, HM modulated MAPK pathways by activating the JNK pathway and by inhibiting ERK phosphorylation. TLR4 receptor downregulation enhanced HM-induced apoptosis while TLR4 receptor blockade partially alleviated HM-inhibited ERK phosphorylation.

**Conclusion:**

Altogether, these findings indicate that HM exerts pro-apoptotic effects and inhibits MAPK pathway through TLR4 in mCRC and colorectal adenocarcinoma cells, suggesting HM as a promising natural-based drug for the treatment of colorectal cancer.

## Background

Colorectal cancer (CRC) is the fourth deadliest cancer type in men and the second in women worldwide [1]. The primary cause of most CRC treatment failure is mainly metastatic CRC (mCRC) cells and subsequent metastatic disease [2, 3]. Conventional chemotherapy for both colorectal adenocarcinoma and mCRC involves highly toxic drugs and is associated with serious and undesirable side effects [4]. Targeted therapy employs small molecule-based drugs or monoclonal antibodies raised against abnormally expressed proteins that are crucial for colorectal adenocarcinoma and mCRC cell growth, including anti-epidermal growth factor receptor (EGFR) agents, e.g. panitumumab and cetuximab, in addition to anti-vascular endothelial growth factor (VEGF) agents, e.g. bevacizumab and aflibercept [5–7]. As chemotherapy induces toxic effects and targeted therapy is very expensive, thus it is necessary to develop novel therapeutic drugs that might eliminate advanced colorectal adenocarcinoma and mCRC cells [8, 9].

Numerous studies have reported the anticancer effects of herbal medicines or phytochemicals for the treatment of CRC. Herbal medicines may have improved efficacy and safety compared to other regimens as well as the potential to be used as a complementary therapy for advanced colorectal adenocarcinoma and mCRC [10–12]. Examples of current clinically used phytochemicals include Catharanthus alkaloids (Vinca rosea) [13] and Camptotheca (happy tree, cancer tree) [14], while others are currently in clinical trials such as curcumin, green tea and soybeans [15]. In vitro studies on different CRC cell lines demonstrated the anti-proliferative effects of phytochemicals, including green tea extracts [16], soybeans [17], garlic [18] or Chinese gold thread [19]. Cell growth inhibition and apoptosis induction may result from increased reactive oxygen species (ROS), activation of adenosine monophosphate (AMP)-activated protein kinase with VEGF reduction, inhibition of insulin-like growth factor-I receptor signaling, or NF-κB pathway inactivation [16–19].

*Nigella sativa* (*N. sativa*) or black cumin exhibits anticancer properties including anti-proliferative, pro-apoptotic, anti-mutagenic, and anti-metastatic effects against neoplasms including leukemia, as well as breast, colon, pancreatic, hepatic, lung, skin, renal, prostate and cervical cancers [20, 21]. Melanins are natural dark biological pigments produced by animals, plants and microorganisms that mediate apoptosis both in vitro [22] and in vivo [23]. Different apoptotic pathways including p53, the APE/Ref-1-p53 and caspase-3-independent pathways have been suggested to be induced by melanin [24]. Herbal Melanin (HM) is an extract component of the seed coats of *N. sativa* which has immuno-modulatory and anti-ulcer properties. It acts through transmembrane toll-like receptor (TLR)4 [25–28]. TLR4 is expressed in immune cells and in various cancer cells including colorectal adenocarcinoma and mCRC [29–32]. Hence, TLR4 has become a target in colorectal cancer therapy due to its critical roles in promoting cancer cell survival, development and progression [33–35]. Furthermore, HM has been demonstrated to induce the cleavage of pro-apoptotic caspase 8 following TLR4 activation [27].

In the present study, HM effect was evaluated for its effects on the proliferation of human colorectal adenocarcinoma cell line HT29 and metastatic mCRC cell line SW620. We showed that HM exerted anti-proliferative effects on both CRC cell subtypes. An increase in ROS production and a decrease of glutathione levels in both HM-treated CRC cell sub-types were also observed. Hence, HM induced (i) ROS-mediated apoptosis, (ii) altered the expression of Bcl2 family anti-apoptotic proteins, enhanced cytochrome c release associated with increased mitochondrial outer membrane permeability, activated caspase cascade, and (iii) modulated MAPK pathways in human CRC cells resulting in cell death process. After TLR4 blockade, we also demonstrated that TLR4 was partially involved in HM-inhibited ERK phosphorylation. These findings support the hypothesis that HM may be effective for the treatment of advanced colorectal adenocarcinoma and mCRC.

## Materials and methods

### Reagents

All reagents were obtained from Sigma-Aldrich unless otherwise mentioned.

### Cell culture

Human colorectal adenocarcinoma HT29 and metastatic colorectal cancer (mCRC) SW620 cell lines were obtained from American Type Culture Collection (ATCC, Manassas VA, USA) and grown in DMEM (Invitrogen, by Thermo Fischer Scientific, Eugene, OR, USA) supplemented with 10% heat-inactivated fetal bovine serum (FBS, Thermo Fischer Scientific), 100 μg/ml streptomycin, 100 IU/ml penicillin and 2 mmol/l l-glutamine. Cells were cultured at 37 °C in a saturated air humidity/5% CO_2_-incubator. At confluence, the cells were passaged every 2–3 days using enzymatic digestion with 0.05% trypsin/0.02% EDTA and split at a ratio of 1:2 or 1:3. Throughout the study, the cells were used between passages 5 and 9.

### Extraction and preparation of HM

HM was extracted, verified by physicochemical methods and prepared for use as previously reported [26]. Briefly, we used the alkali solubilization and acid aggregation of melanin from the seed coats of *N. sativa* which were purified by centrifugation and filtration, then vacuum dried. A solution at a concentration of 1 g/l of the lyophilized HM was prepared by dissolving in 1 N NaOH, followed by pH adjustment to 7.0 and filtration through 0.22 μm filters. A stock solution of HM was prepared at concentrations of 0.1–1 g/l in sterile distilled water for further experimental usage. No endotoxin was detected in HM solution (< 0.125 EU/ml detection limit).

### Cell viability assay

Cell viability was determined using MTT assay as previously described [36]. Briefly, the cells (5 × 10^3^) were seeded in a 96-well plate (Corning, NY, USA) in complete medium. After 24 h of incubation, the cells were untreated (considered as the control) or treated with HM at various concentrations (5–200 μg/ml) for 24 h of incubation. Freshly prepared 10 μl of 3-(4, 5-dimethylthiazol-2-yl)-2,5-diphenyltetrazolium bromide MTT (5 mM) solution were added to the cells and further incubated for 2 h. Thereafter, 100 μl of dimethyl sulfoxide (DMSO) were added in each well and the crystals were dissolved through careful pipetting. The absorbance of the product was measured at 540 nm using a Synergy™ 2 multi-mode microplate reader (Biotech, VA, USA). The experiments were performed in triplicate for each condition.

### Cytotoxicity assay

The cell seeding density for CRC cell proliferation was optimized using colorectal adenocarcinoma cell line HT29. Briefly, HT29 cells (5 × 10^3^ cells in 150 µl medium/well) were seeded in 16-well E-plates (ACEA Biosciences Inc, San Diego, CA, USA) according to the xCELLigence Real Time Cell Analyzer (RTCA) DP manufacturer’s instructions. The following day, HM (50–100 µg/ml) was added to the cells for 48 h of incubation. Baseline cell indices were calculated using RTCA software (ACEA Biosciences) for at least two measurements based on three replicate experiments.

### Cellular reactive oxygen species (ROS) fluorometric detection assay

Cellular reactive oxygen species (ROS) generated was monitored in HT29 and SW620 cells using the non-fluorescent H2DCFDA reagent that converts into highly fluorescent 2′,7′-dichlorofluorescein (DCFDA) when reacts with several ROS including hydrogen peroxide (H_2_O_2_) [37]. In brief, 5000 cells/well were seeded with phenol red free-DMEM in a 96-well microplate. The cells were untreated or treated with different concentrations (50–100–200 μg/ml) of HM. After 24 h of incubation, DCFDA was added to the wells at 5 μM for 30 min. Using Epoch fluorescence microplate reader (Biotech, Winooski, VT, USA), fluorescence intensity was measured at excitation and emission wavelengths of 485 and 535 nm, respectively. For shorter incubation times, cellular ROS generation was also determined using flow cytometry. Briefly, cells were cultured and treated as described above. After 1 h of cell treatment with (100 μg/ml) HM, the cells were exposed to 5 μM DCFDA for 30 min. The cells were washed twice with 1 × PBS and then analyzed on Becton–Dickinson (BD) FACScalibur flow cytometer using Cell Quest Pro software (BD Biosciences, San Jose, CA, USA) for ROS detection using the 488 nm laser for excitation and detected at 535 nm (FL1 channel).

### Glutathione colorimetric detection assay

Total glutathione levels were measured using a glutathione assay kit (Cayman Chemical Co. Ann Arbor, MI, USA). Cells were treated with different concentrations of HM and harvested after 24 h of incubation. The cell pellets were homogenized in 50 mM MES buffer containing 1 mM EDTA and centrifuged at 10,000×*g* for 15 min at 4 °C. The supernatants were mixed with assay cocktail along with standards in 96-well plates and incubated for 25 min. The absorbance was measured using the end-point method at 405 nm.

### Western blotting

Whole cell lysates were prepared using radioimmunoprecipitation assay (RIPA) lysis buffer (Boston Bio products, Ashland, MA, USA) and protein concentration was determined using the Bradford Protein reagent (Bio-Rad laboratories Inc., Hercules, CA, USA). Equal amounts of cell lysate proteins were loaded and electrophoresed using 4–20% Mini-Protean TGX precast gels (Bio-Rad) and subsequently transferred to a 0.22 μm nitrocellulose transfer membrane using the trans-blot turbo transfer system (Bio-Rad). The membranes were blocked in 5% skimmed milk in PBS containing 0.1% Tween-20 (PBST) for 1 h at room temperature and were incubated overnight at 4 °C with the following primary antibodies directed against: cytochrome c (1:200 dilution) purchased from Abcam (Cambridge, UK), Bcl2, Bcl-xL, Bad, phospho-JNK (p-JNK), p-ERK (1:1000 dilution) and β-actin (1:10,000) from Santa Cruz Biotechnology (Dallas, TX, USA), p–c-JUN and activation transcription factor (ATF)2 (1:1000 dilution) from Cell Signaling Technology Inc. (Danvers, MA, USA). Immuno-reactivity was occurred after incubation with horseradish peroxidase-conjugated secondary antibodies for chemiluminescence detection using Clarity Western ECL Substrate (Bio-Rad). Images were captured by C-DiGit™ Blot Scanner (LI-COR Biosciences, Lincoln, NE, USA) analyzed using Image Studio™ software (LI-COR Biosciences).

### Apoptosis fluorometric determination assays

Both HT29 and SW620 cell lines were treated with HM at 100 µg/ml, concentration at which HM exerted strong anti-proliferative effects. Cells were harvested by trypsinization after 6 and 24 h of incubation. Detection of apoptosis was assessed by exposing the cells to Green Apopxin and 7-aminoactinomycin D (7-AAD) components (Apoptosis/Necrosis Detection kit, Abcam) according to the manufacturer’s instructions. Acquisition and analysis of the stained cells was performed on BD FACScalibur flow cytometer in which the fluorescence emission was measured at 530 nm (FL1 channel) for Green Apopxin and at a minimum of 575 nm (FL3 Channel) for 7-AAD where 10,000 events were gated for each test. The effect of HM on HT29 cells was also investigated in the presence or absence of the anti-oxidative agent *N*-acetylcysteine (NAC, Santa Cruz Biotechnology), which is known to inhibit apoptosis in a ROS-dependent manner. Briefly, the cells were incubated with 5 mM of NAC solution for 30 min followed by treatment with 100 μg/ml of HM. The cells were incubated for 24 h then the apoptosis levels were measured using FITC Annexin V/Dead Cell Apoptosis Kit (Molecular Probes by ThermoFischer Scientific, Eugene, OR, USA) on BD FACScalibur flow cytometer at 530 nm (FL1 channel) for FITC Annexin V and at a minimum of 575 nm (FL3 Channel) for propidium iodide (PI). For each test, 10,000 events were gated and analyzed using BD Cell Quest Pro software.

In order to visualize the increased permeability of the mitochondrial outer membrane allowing cytochrome c release in HT29 and SW620 cells, the cells were seeded in 8-well slide chambers and treated with HM as described above. Mitochondrial outer membrane permeability was determined using Image-iT^®^ LIVE Mitochondrial Transition Pore Assay Kit according to the manufacturer’s instructions (Molecular Probes, Life technologies, Carlsbad, CA, USA). Mitochondrial transition pore activity was deemed positive if cells displayed quenching mitochondrial calcein green fluorescence (excitation 494/emission 517 nm) caused by the cobalt entered inside the mitochondria (stained with MitoTracker^®^ Red dye, excitation 579/emission 599 nm), visualized using Leica TCS SP8 fluorescence microscope system (Leica Biosystems, Wetzlar, Germany).

### Caspase-3/-7 Assay fluorometric detection assay

Cells were cultured and treated as described above. To measure the caspase cascade activation, we used the Vybrant™ FAM Caspase-3/-7 Assay Kit (Invitrogen). Briefly, the cells were stained with FLICA reagent and incubated for 60 min at 37 °C then the cells were washed twice with phosphate buffered saline (PBS) before PI was added and incubated for 5–10 min at room temperature. The cells were analyzed using a BD FACScalibur flow cytometer with 488 nm excitation and green emission for the FLICA-stained cells (FL1) or red emission for PI (FL3).

### TLR4 receptor downregulation and neutralization assays

To investigate whether HM-induced apoptosis and HM-inhibited ERK phosphorylation act through TLR4 receptor, siRNA technology and neutralizing antibody were applied for TLR4 downregulation and receptor blockade.

HT-29 and SW620 cells were grown to reach 50% of confluence. Next day, Lipofectamine transfection reagent RNAi/Max, negative control siRNA and TLR4 siRNA (Santa Cruz Biotechnology) were diluted in Opti-MEM medium (Thermo Fisher Scientific, Waltham, MA, USA). Diluted siRNAs and Lipofectamine RNAi/Max reagent were mixed together (1:1 ratio) and incubated for 30 min. Complete medium was removed and siRNA-lipid complex was added dropwise to the cells. Cells were transfected for 48 h at 37 °C and then HM (100 μg/ml) was added for a further 24 h of incubation. TLR4 siRNA-transfected cells were harvested for TLR4 expression assessment using Western blot and for apoptosis analysis using FACS.

In order to neutralize TLR4 receptor, both HT29 and SW620 cells were treated with 20 μg/ml rabbit polyclonal anti-TLR4 antibody (Abcam, concentration fixed from pilot studies) along with 20 μg/ml IgG (Abcam). After 2 h of incubation, the cells were treated with 100 μg/ml of HM for 24 h incubation followed by apoptosis analysis and ERK phosphorylation assessment using Western blot analysis.

### Statistical analysis

The results are expressed as the mean ± standard deviation (SD). Data points are collected for a minimum of three independent experiments. One-way ANOVA test was used to compare two groups and a value of p < 0.05 considered significant.

## Results

### HM inhibits colorectal adenocarcinoma and mCRC cell proliferation

**Fig. 1:**
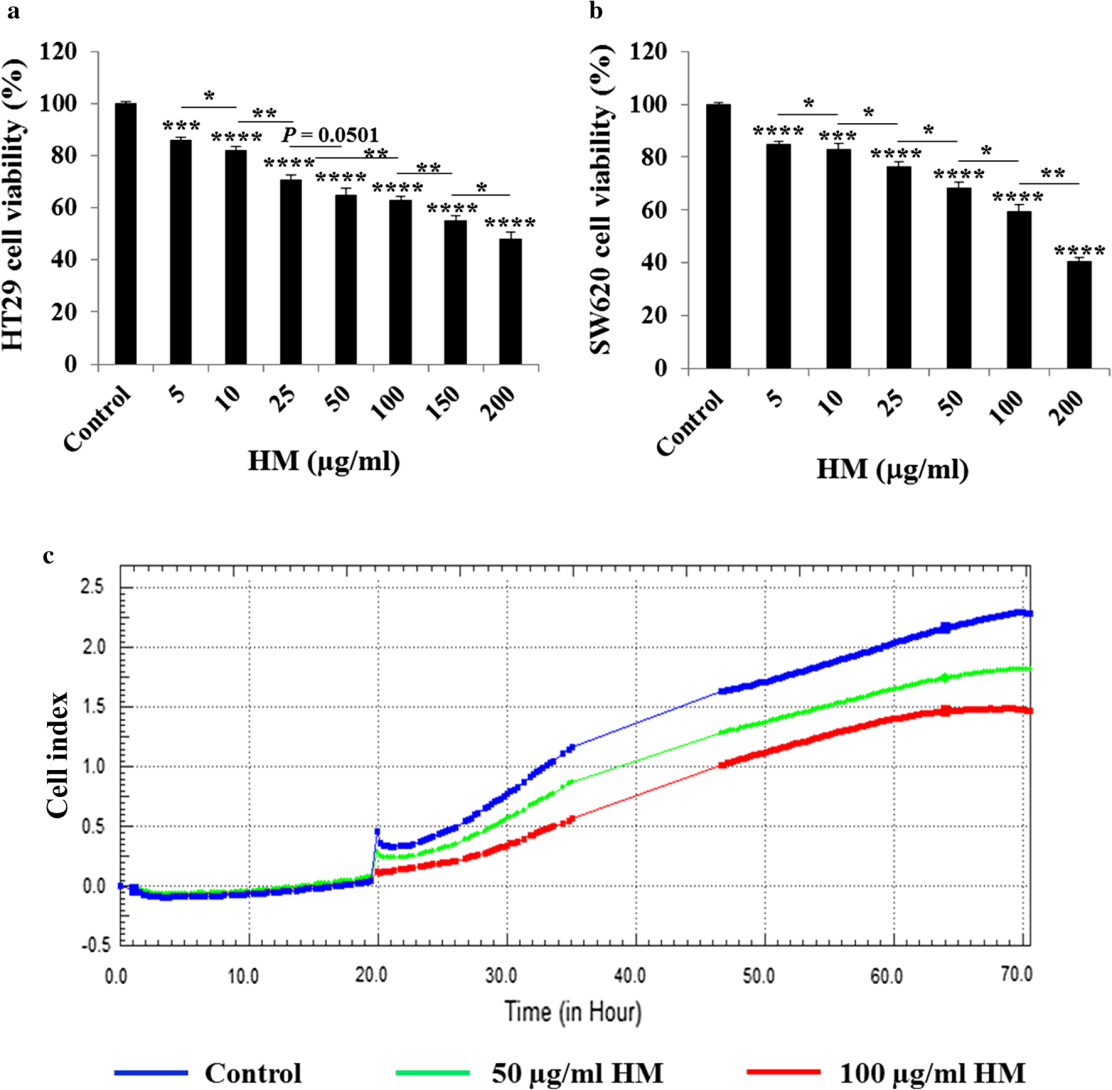
HM inhibits colorectal adenocarcinoma HT29 and mCRC SW620 cell viability and proliferation. Cell viability was determined using MTT assay in HT29 (**a**) and SW620 (**b**) CRC cells that were cultured in 96-well plates for 24 h prior to treatment with various concentrations (5–200 μg/ml) of HM. The inhibitory effect of HM on CRC cell viability was observed after 24 h of treatment. The bar graphs show the percentage viability related to the control (corresponding to 100%) and the results are presented as mean ± standard deviation (SD) of three independent experiments. (*), (**), (***), and (****) signify a statistically significant difference (*P* < 0.05, *P* < 0.01, *P* < 0.001, and *P* < 0.0001) compared with the control. **c** The anti-proliferative effect of HM on HT29 cells was recorded using xCelligence RTDP for cell number measurement in real-time

Colorectal adenocarcinoma HT29 and mCRC SW620 cells were treated with various concentrations (5–200 μg/ml) of HM for 24 h and a dose-dependent inhibition of the cell viability was assessed in each cell line, in comparison with high cell viability of untreated cells (Fig. 1a, b). Of note, no significant difference was observed comparing the HM-inhibited viability of HT29 cells exposed to 50 μg/ml of HM with HM-inhibited viability of HT29 cells exposed to 100 μg/ml of HM (Fig. 1a). However, using xCELLigence RTDP in order to measure the cell proliferation in real-time based on the cell number, an anti-proliferative effect of HM on colorectal adenocarcinoma HT29 cells treated with HM at 50 and 100 μg/ml was clearly recorded (Fig. 1c). In comparison to the untreated cell growth, HM inhibited HT29 cell proliferation in a dose-dependent manner (Fig. 1c).

### HM alters redox balance in both CRC sub-types

**Fig. 2:**
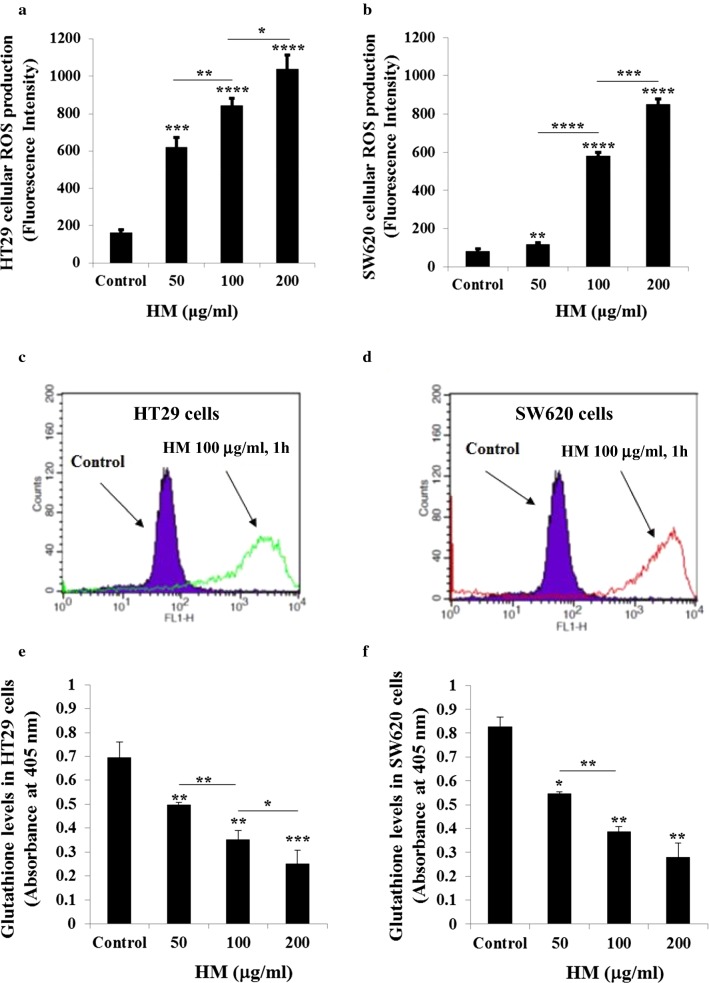
HM alters redox balance in CRC cells. HM-treated HT-29 (**a**) and SW620 (**b**) CRC cells were incubated with c-H2-DCFDA for 30 min and the fluorescence of the oxidized DCF was measured using a fluorescence plate reader. The bar graphs (**a**, **b**) show the fluorescence intensity and the results are presented as mean ± SD of three independent experiments. **c** Histograms showing ROS detected in HT29 (**c**) and SW620 (**d**) CRC cells after 24 h of treatment with HM (100 μg/ml). Total glutathione levels were detected in HT29 (**e**) and SW620 (**f**) cells after 24 h of treatment with HM in a dose-dependent manner. The bar graphs (**e**, **f**) show the absorbance read at 405 nm and the results are presented as mean ± SD of three independent experiments. (*), (**), (***), and (****) signify a statistically significant difference (*P* < 0.05, *P* < 0.01, *P* < 0.001, and *P* < 0.0001) compared with the control, the untreated cells

Many current anticancer agents inhibit cell viability by increasing cellular reactive oxygen species (ROS) production and depleting glutathione (GSH) levels, leading to accumulation of cellular ROS causing apoptosis and cell death. This is in contrast to physiological situations in which cellular redox balance is maintained [37, 38]. The treatment of HT29 cells with HM resulted in increased generation of cellular ROS in a dose-dependent manner (Fig. 2a). Similar results were obtained in HM-treated mCRC cells SW620 (Fig. 2b). These findings were confirmed using flow cytometry (Fig. 2c, d). HT29 and SW620 cell exposure to HM resulted in depletion of total GSH levels (Fig. 2e, f) under the same conditions in which increased of generated cellular ROS levels were observed (Fig. 2a, b).

### HM induces ROS-mediated apoptosis in both CRC sub-types

**Fig. 3:**
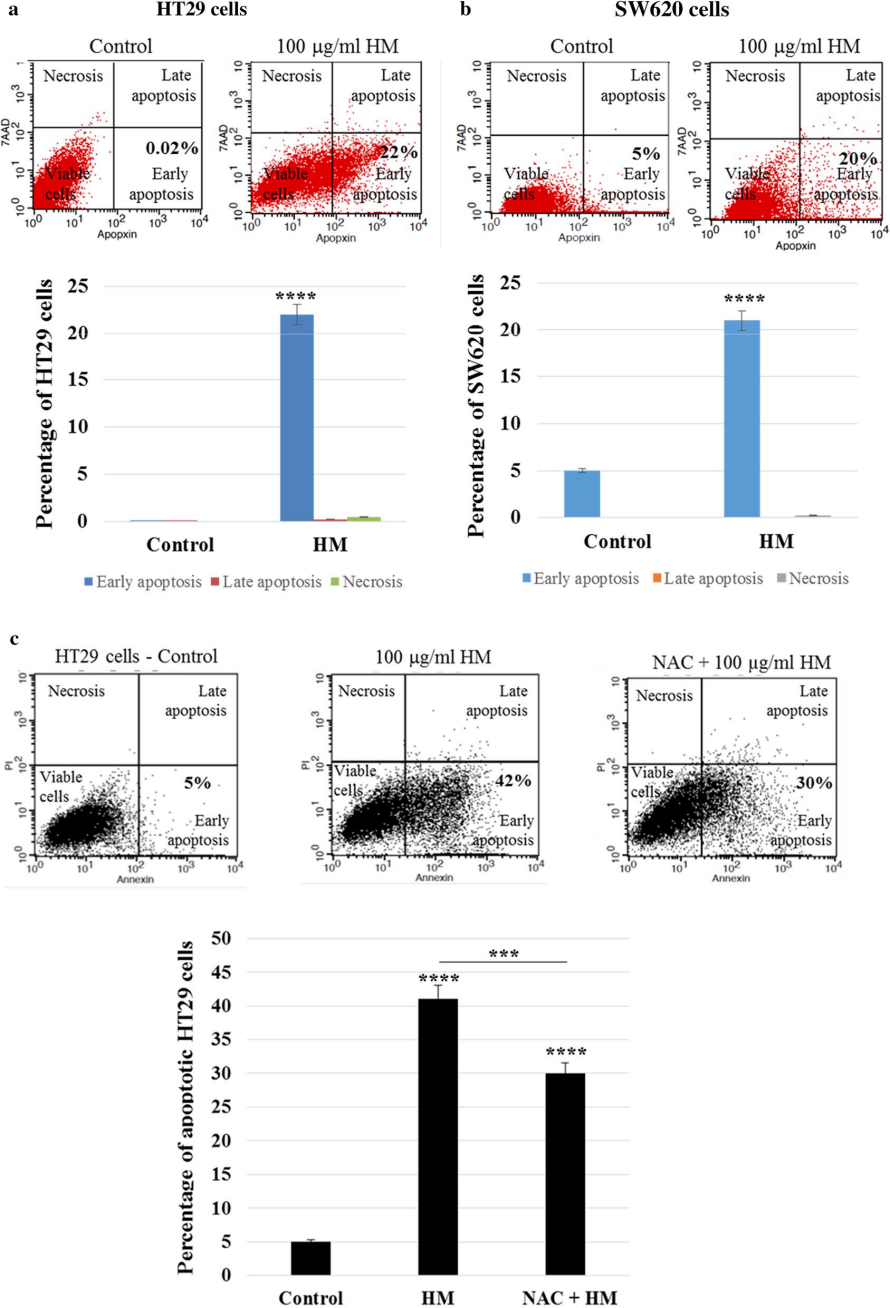
HM induces ROS-mediated apoptosis in CRC cells. Representative scatter plots showing the percentage of apoptotic cells (lower right) measured using Apopxin in the FL1 channel versus 7-AAD in FL3 after 24 h of treatment with HM tested at 100 µg/ml on HT29 (**a**) and SW620 (**b**) cells in comparison to the control, the untreated cells. (**c**) Effect of NAC (ROS inhibitor) on HT29 cell apoptosis induced by 100 µg/ml of HM after 24 h of exposure. The bar graph shows the percentage of apoptotic cells and the results are presented as mean ± SD of three independent experiments. (***) and (****) signify a statistically significant difference (*P* < 0.001 and *P* < 0.0001) compared with the control

To determine whether HM treatment induces apoptosis or necrosis in both CRC sub-types, HT29 and SW620 cells were treated with HM at 100 μg/ml (concentration showing a strong anti-proliferative effect) for 24 h. Using flow cytometry, HM treatment resulted in a marked increase of the apoptotic cells as compared to healthy untreated CRC cells (Fig. 3a, b). Close to 25% of the HM-treated cell populations were Annexin V-positive and 7AAD-negative, indicating that HM induced apoptosis and not necrosis (Fig. 3a, b). The results from 7-AAD used as a nucleic acid dye were null for all conditions, indicating the absence of necrosis. The cell pre-treatment with N-acetylcysteine (NAC), which acts as an antioxidant and also known as an ROS inhibitor [39], alleviated the effect of HM on apoptosis, confirming that HM-mediated apoptosis is partially dependent on ROS generation (Fig. 3c).

### HM inhibits Bcl2 family proteins, induces cytochrome c, and increases mitochondrial outer membrane permeability

**Fig. 4:**
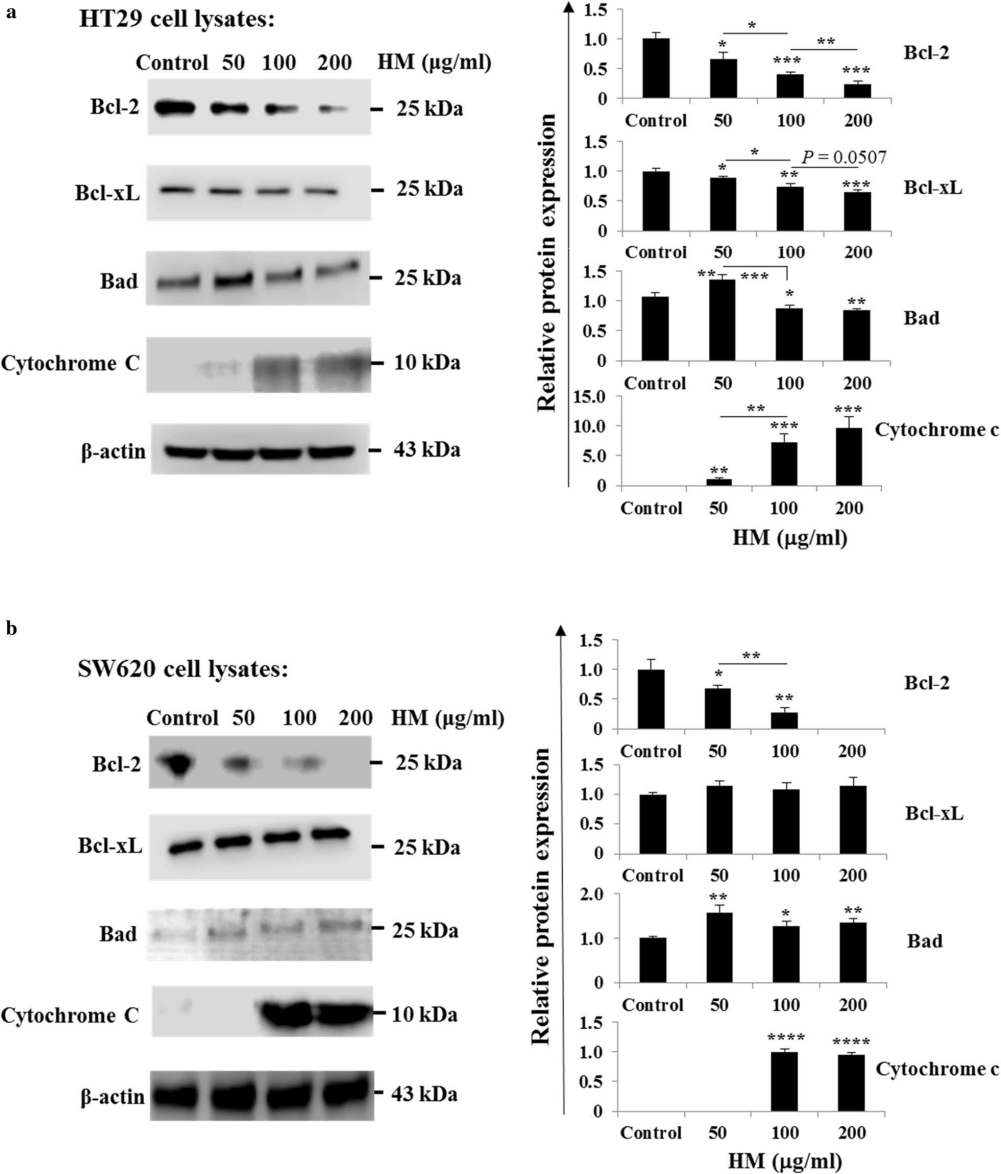
HM inhibits Bcl2 family proteins and induces cytochrome c in CRC cells. The effect of HM on Bcl2 family proteins was investigated using Western blot technology. Untreated and HM-treated HT29 (**a**) and SW620 (**b**) cell lysates were immunoblotted with the antibodies directed against the indicated proteins. Bar graphs showing the Bcl2 protein expression relative to the basal expression detected in control cells given an arbitrary value of 1.0 using β-actin as the loading control. All data present the mean + SD of three independent experiments. (*), (**), and (***) signify a statistically significant difference (*P* < 0.05, *P* < 0.01, and *P* < 0.001) compared with the control

**Fig. 5:**
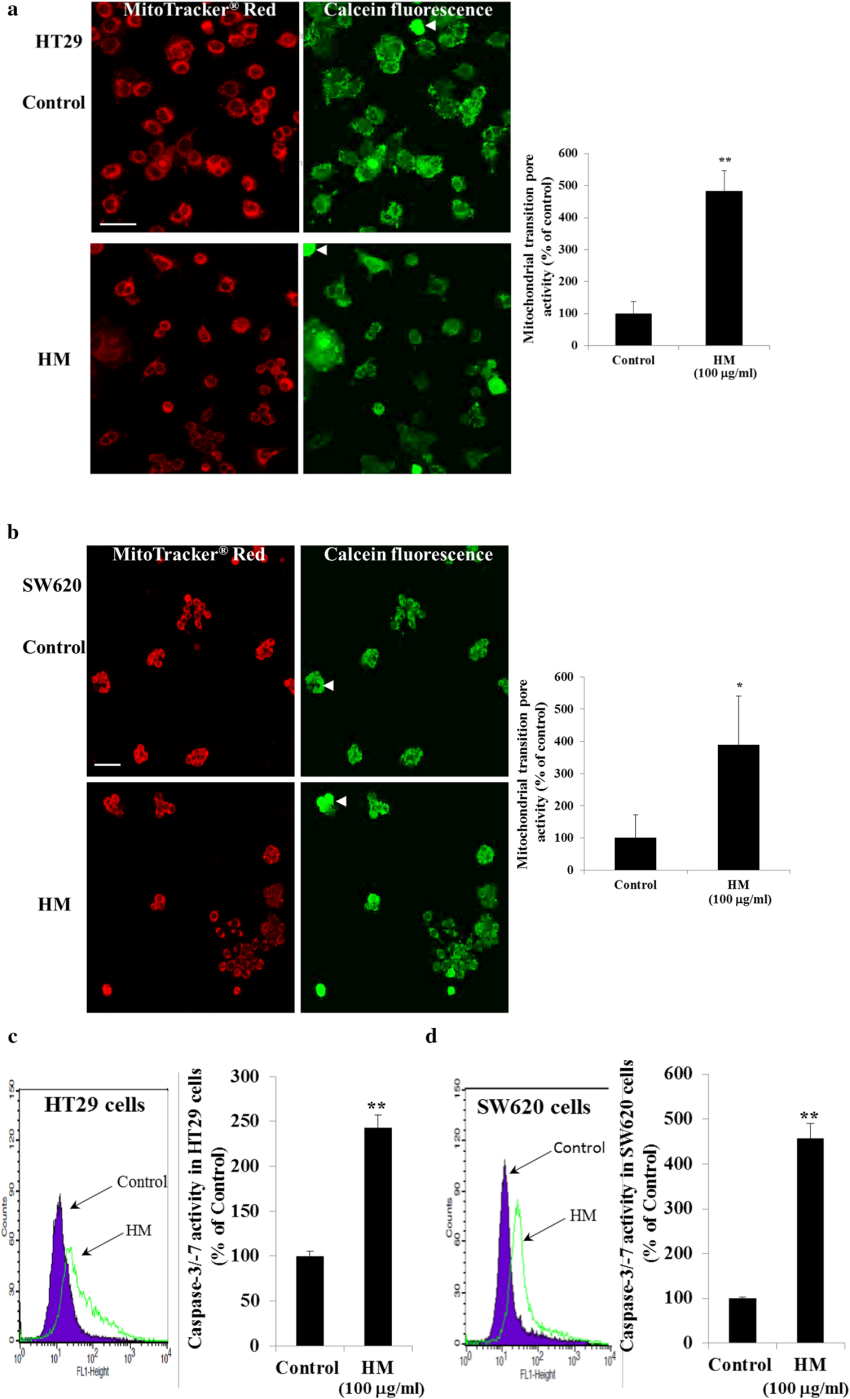
HM increases mitochondrial outer membrane permeability and activates executioner caspase-3/-7 in CRC cells. Representative photomicrographs (scale bar = 50 μm) showing the induction of apoptosis in HT29 (**a**) and SW620 (**b**) untreated cells (control) and (100 μg/ml) HM-treated cells at 24 h post-treatment through increased permeability of the mitochondrial outer membrane as indicated by the quenched green fluorescence of calcein occurred in whole the apoptotic cells (examples pointed by arrows). Mitochondria were stained with Mitotracker Red CMXRos™ dye for whole cell density visualization. Bar graphs showing the mitochondrial transition pore activity normalized to the control. The results expressed in percentage are presented as mean ± SD of at least four independent experiments. Representative histograms showing the increase of cleaved caspase-3 and caspase-7 detected in HM-treated HT29 (**c**) and SW620 (**d**) CRC cells. Respective bar graphs summarizing the caspase-3/-7 activity normalized to the control. The results expressed in percentage are presented as mean ± SD of three independent experiments. (*) and (**) signify a statistically significant difference (*P* < 0.05 and *P* < 0.01) compared with the control

Bcl2 family proteins are known to be involved in apoptosis, cell proliferation, and cancer cell invasion and metastasis [40–42]. The balance between pro-apoptotic (Bax, Bak) and anti-apoptotic proteins (Bcl2, Bcl-xL) determines the cell fate towards cell survival or cell death under stress [43]. Treatment of HT29 cells with HM resulted in the inhibition of Bcl2 expression in a dose-dependent manner (Fig. 4a) and inhibition of Bcl-xL expression to a lesser extent. While HM also inhibited Bcl2 expression in SW620 cells (Fig. 4b), HM had no effect on Bcl-xL expression. In both CRC subtypes, a significant increase of Bad expression was detected after cell treatment with 50 μg/ml of HM (Fig. 4a, b). At higher concentration (100–200 μg/ml), HM inhibited Bad expression in HT29 cells (Fig. 4a) while Bad expression remained slightly increased in SW620 cells (Fig. 4b). Initiation of apoptosis signaling activates Bax which binds to mitochondrial outer membrane and induces the opening of voltage-dependent anion channel, VDAC leading to cytochrome c release from mitochondria into the cytosol where it activates the caspase cascade [41, 44]. Treatment of HT29 cells with HM resulted in the increased expression of cytochrome c (Fig. 4a). Similar results were obtained using SW620 cells (Fig. 4b). Mitochondrial transition pore opening activity that allows cytochrome c release was also visualized in both CRC sub-types in the presence or absence of 100 μg/ml of HM using fluorescence microscopy. Representative photomicrographs showed that HM increased mitochondrial outer membrane permeability of both HT29 and SW620 cells as indicated by the green fluorescence emission from the mitochondrial calcein dye occurred in whole the apoptotic cells (Fig. 5a, b). To check whether executioner caspases were activated in the induction of apoptosis, HM-treated HT29 cells were incubated with caspase-3 and -7 substrates. HM was determined to activate caspase-3 and caspase-7 in HT29 cells as depicted by peak shifts (Fig. 5c). Similar findings of the increased of caspase-3/-7 activity were observed in HM-treated SW620 cells as compared to their caspase activity detected in the control, untreated cells (Fig. 5d). Thus these results indicate that HM induces apoptosis through intrinsic mitochondrial-dependent pathways.

### HM regulates MAPK pathways in both CRC sub-types

**Fig. 6:**
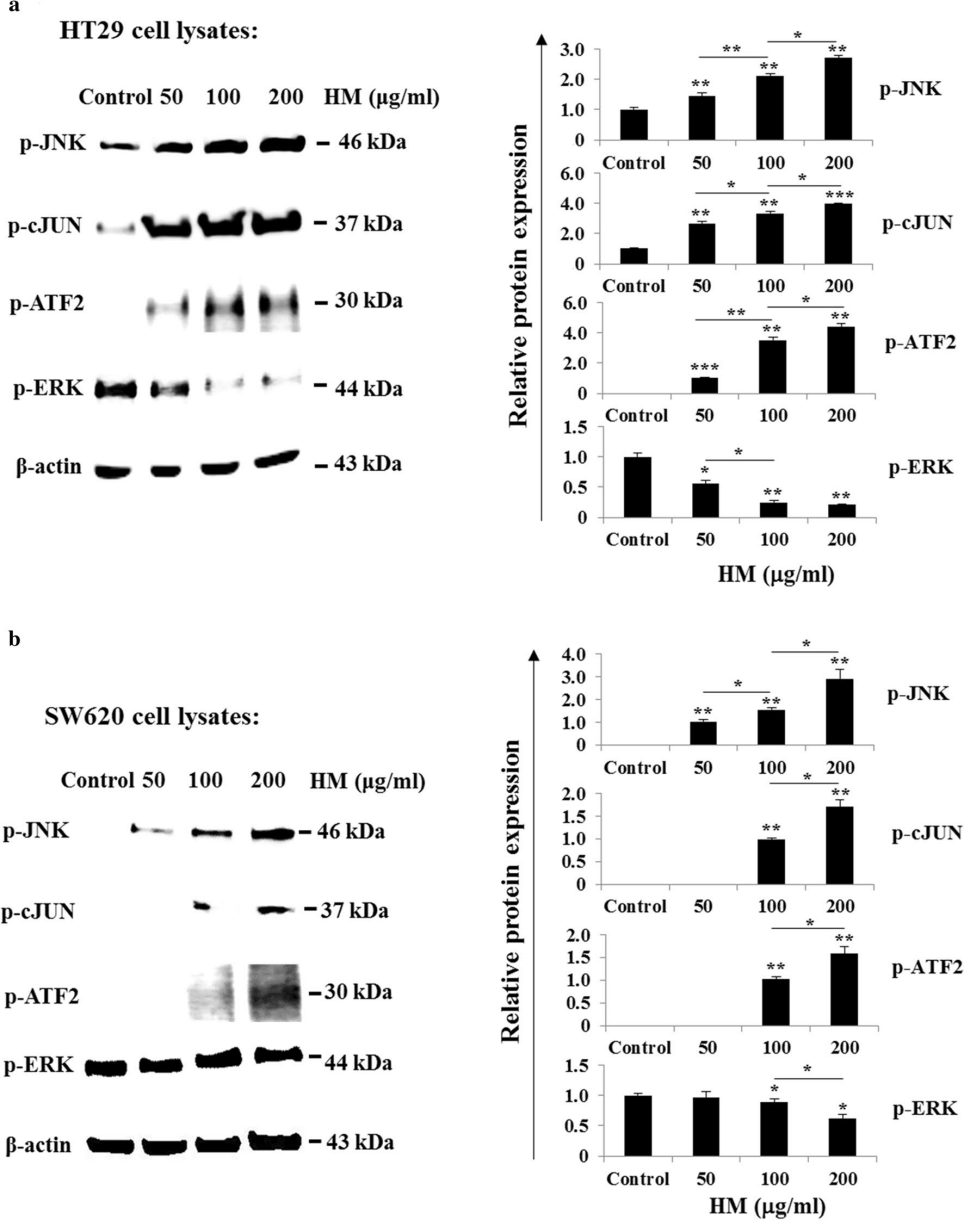
HM Regulates MAPK pathways in CRC cells. The effect of HM (100 μg/ml) on MAPK kinases/ERK signaling pathway was evaluated by Western blotting based on the expression of their phosphorylated forms. Both HM-treated HT29 (**a**) and SW620 (**b**) cell lysates were immunoblotted with the antibodies directed against the indicated proteins. Bar graphs showing the phosphorylation of MAPK and ERK relative to the basal phospho-protein expression detected in control cells given an arbitrary value of 1.0 using β-actin as the loading control. All data present the mean + SD of three independent experiments. (*), (**), and (***) signify a statistically significant difference (*P* < 0.05, *P* < 0.01, and *P* < 0.001) compared with the control

To decipher how HM affects cellular signaling in both colorectal adenocarcinoma HT29 and mCRC SW620 cells, the effect of HM on certain MAPK expression detected in these cell lines was assessed using Western blot. JNK is known to regulate ROS-mediated apoptosis or cell death while ERK is known to be involved in cancer initiation and progression [45, 46]. Both HT29 and SW620 cell treatment with various concentrations (50–100–200 μg/ml) of HM resulted in a marked increase in JNK phosphorylation in a dose-dependent manner (Fig. 6a, b). To confirm this finding, the effects of HM on downstream JNK target genes was determined, and as expected HM enhanced the phosphorylation of cJun and ATF2 in HT29 cells (Fig. 6a) and in SW620 cells with an exposure higher than 100 μg/ml of HM (Fig. 6b). In addition, HM inhibited ERK phosphorylation in a dose-dependent manner in both CRC subtypes, as compared to the basal level of ERK phosphorylation (p-ERK) detected in untreated cells (Fig. 6a, b). Thus, these findings indicate that HM activates the JNK pathway while inhibits ERK phosphorylation in colorectal adenocarcinoma and mCRC cells.

### HM-inhibited ERK phosphorylation acts partially through TLR4

**Fig. 7:**
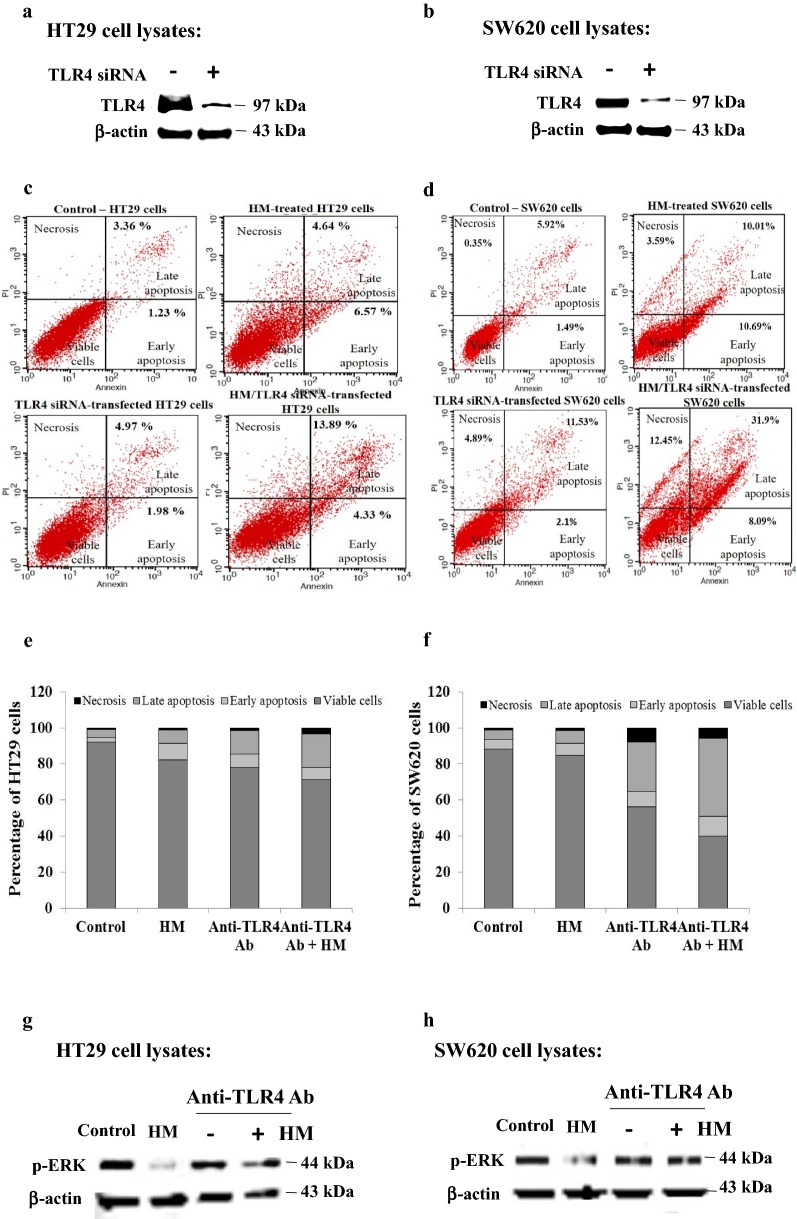
Both TLR4 downregulation and TLR4 blockade enhance HM-induced apoptosis while TLR4 blockade reduce HM-inhibited ERK phosphorylation. Representative Western blots showing downregulation of TLR4 expression detected in TLR4 siRNA-transfected HT29 (**a**) and SW620 (B) cells as compared with basal TLR4 expression level in untransfected cells (**a**, **b**). Representative scatter plots showing the percentage of apoptotic and necrotic cells in control untreated cells (top left), HM-treated cells (top right), TLR4 siRNA-transfected cells (bottom left), and in HM-treated TLR4 siRNA-transfected cells (bottom right) using HT29 (**c**) and SW620 (D) cells. Bar graphs showing the distribution of the percentage viable, early apoptotic, late apoptotic and necrotic cells in untreated cells, HM-, anti-TLR4 antibody (Ab)-, and HM added to anti-TLR4 Ab-treated HT29 (**e**) and SW620 (**f**) cells. Representative Western blot showing the expression of phospho-ERK (p-ERK) in untreated cells, HM-, anti-TLR4 antibody (Ab)-, and HM added to anti-TLR4 Ab-treated HT29 (**g**) and SW620 (**h**) cells

To verify whether TLR4 plays a key role in HM anticancer activities including apoptosis induction and inhibition of ERK phosphorylation in colorectal cancer cells, TLR4 expression was downregulated using siRNA technology and its receptor was neutralized using neutralizing antibody. Using Western blot, a concomitant decrease of TLR4 expression was observed in both TLR4 siRNA-transfected HT29 (Fig. 7a) and SW620 (Fig. 7b) cells, as compared with the basal level of TLR4 expressed in untransfected cells (Fig. 7a, b) and detected in NC siRNA-transfected cells (data not shown). Downregulation of TLR4 expression resulted in a slight induction of apoptosis in both TLR4 siRNA-transfected HT29 (Fig. 7c, bottom left) and SW620 (Fig. 7d, bottom left) cells. An enhancement of TLR4 downregulation-induced apoptosis was observed in both transfected HT29 (Fig. 7c, bottom right) and SW620 (Fig. 7d, bottom right) cells by the addition of (100 μg/ml) HM, as compared with HM-induced apoptosis in untransfected cells (Figs. 7c, 7d, top right). TLR4 blockade using neutralizing anti-TLR4 antibody confirmed this induction of apoptosis in both colorectal cancer HT29 (Fig. 7e) and SW620 (Fig. 7f) cells and revealed to be enhanced by the presence of HM, as compared with untreated cells, the control (Figs. 7e, f). In regards to HM-inhibited ERK phosphorylation, TLR4 blockade reduced the inhibitory effect of HM on ERK phosphorylation detected in colorectal adenocarcinoma HT29 (Fig. 7g) and mCRC SW620 (Fig. 7h), as compared to HM-inhibited ERK phosphorylation detected in both cells (Figs. 7g, h).

## Discussion

Historically, medicinal plants have been used to treat various human diseases including cancer [47]. *Nigella sativa* (also known as black cumin) is a member of the Ranunculaceae family well known to have pharmacological properties and immunoprotective effects as reported in several studies [48]. Herbal melanin (HM) extract from N*igella sativa* seeds has been identified as a ligand for TLR4 that activates NF-κB signaling pathway [25–27]. Previous studies have suggested the use of various TLR4 ligands as potential anticancer agents [49, 50]. In the current study, we tested HM on HT-29, a colon adenocarcinoma cell line, and SW620, a metastatic colorectal cancer (mCRC) cell line in order to investigate its potential anticancer effects. In both CRC cell lines, HM inhibited the cell proliferation, increased cellular ROS production, induced mitochondrial-dependent apoptotic pathway and modulated MAPK pathway including inhibition of ERK phosphorylation, demonstrated to partially occur through TLR4. Moreover, HM has shown no cytotoxic effect with quasi absence of necrotic cells in HM-treated apoptotic CRC cell population, suggesting HM as a promising anticancer agent for the treatment of colorectal adenocarcinoma and mCRC.

Most of the anticancer agents inhibit cell viability by increasing ROS production and depleting glutathione (GSH) levels. While ROS is produced through normal oxygen metabolism, increased ROS levels beyond a threshold that can induce cell damage and apoptosis in tumor cells [51]. In the current study HM increased the production of ROS as indicated by fluorescence intensity of the HM-treated HT-29 and SW620 cells. We attributed ROS production to HM/TLR4 activation as both cell lines express TLR4 [52]. Previously, the induction of ROS via TLR4 activation has been demonstrated [53, 54]. Treating the TLR4 negative kidney cell line HEK293 with HM had not effect on ROS production (data not shown). Our assumption could be confirmed by knocking out TLR4 (TLR4 KO) expression in CRC cells in which an absence of ROS production even in HM-treated CRC TLR4 KO cells could be expected.

Moreover, HM induced depletion of GSH levels in both colorectal cancer cell lines HT29 and SW620, indicating that it modulates the redox balance by shifting cell fate towards cell death. A decline in cellular GSH level accompanied by generation of cellular ROS acts as a potential activator of cellular apoptosis [55]. In order to determine the nature of the observed HM-mediated inhibition of proliferation, ROS and GSH modulation in relation to apoptosis, we examined the HM-treated cells for apoptotic status determination by flow cytometry based on Annexin V and 7-AAD double-staining [56]. Our results indicated that HM induces apoptosis without any effect on necrosis in the two cell lines tested. Finally, we investigated the real time HM-mediated apoptotic pathway based on measuring the mitochondrial transition pore opening activity. In HM-treated mCRC and colon adenocarcinoma cells, a green fluorescence emission from the mitochondrial calcein dye occurred in whole the apoptotic cells as indicator of increased mitochondrial outer membrane permeability, suggesting the release of pro-apoptotic mitochondrial proteins including cytochrome c. In addition, using Western blot, a concomitant increase of cytochrome c was detected in both HM-treated CRC cell lines. Altogether, these findings suggest the main involvement of the mitochondrial (intrinsic) pathway in HM-mediated apoptosis in mCRC and colon adenocarcinoma cells.

The B cell lymphoma 2 (Bcl-2) family proteins play an important role in apoptosis as they control the permeability of the mitochondrial outer membrane. Bcl-2 proteins include Bax (also known as Bcl-2-like protein 4) and Bad (Bcl2-associated death promoter) as pro-apoptotic agents and Bcl2, Bcl-xL (B-cell lymphoma-extra-large) as anti-apoptotic agents. The balance between these two groups controls the cell fate and regulates cell viability, proliferation and metastasis [57]. Bcl2 and Bcl-xL inhibit apoptosis by preventing the release of cytochrome c from the mitochondria which leads to caspase activation and apoptosis [58]. Bcl-2 is more expressed in colorectal carcinoma than in ordinary mucosa and associated with CRC development [59, 60]. Similarly, Bcl-xL is strongly up-regulated in human CRC specimens and has a driving role in CRC tumorigenesis and progression [61]. Our results have shown that HM inhibited Bcl-2 and Bcl-xL expression in the human colorectal cancer cell lines. To further confirm our results we tested, in parallel, the pro-apoptotic protein Bad which is involved in initiating apoptosis. Bad forms heterodimers with anti-apoptotic proteins and prevents them from inhibiting apoptosis. HM increased Bad expression at a lower concentration in both HT29 and SW620 CRC cells, and weakly induced Bad expression at higher HM concentrations in mCRC SW620 cells. All these effects on Bcl2 family proteins revealed to activate the release of cytochrome c from mitochondria into the cytosol, resulting in activation of the caspase cascade including activation of executioners caspase-3/7. Our results demonstrated for the first time that HM induces apoptosis through mitochondrial-dependent intrinsic apoptotic pathway in colorectal adenocarcinoma and mCRC cells.

MAPK kinase (MEK)/ERK MAPK signaling pathways play a key role in the initiation, progression of different types of tumors [45, 46]. In a previous study the treatment of SW620 cells with both a MEK inhibitor and resveratrol increased levels of the apoptotic proteins Bax, caspase-3 and caspase-9 but not of Bcl2 protein [62]. High expression of ERK protein participates in the initiation and development of gastro-enteric tumors such as CRC [63]. c-Jun N-terminal kinases (JNKs) are proteins known to regulate apoptosis and could be activated by various environmental stresses such as DNA-damaging agents and chemo-preventive drugs [64]. The transcription factors c-Jun and ATF-2 are known to be activated by phosphorylation induced by JNKs. In the present study, HM modulated the MAPK pathways through a significant increase in JNK phosphorylation and its downstream substrates (i.e. c-Jun and ATF2) while a decrease of ERK phosphorylation levels was observed in HM-treated CRC cells. All these data suggest that HM may play important role in inhibiting CRC development and progression by modulating MAPK pathways.

Lately, HM has been widely demonstrated to exert immunogenic activities through TLR4 receptor in human acute monocytic leukemia cell line THP-1 [25, 27]. Thus, in this present study, we verified whether TLR4 plays a key role in HM anticancer effects in colorectal adenocarcinoma and mCRC. Our findings showed that the downregulation of TLR4 expression did not impede HM-induced apoptosis but in contrast, induced apoptosis and enhanced HM pro-apoptotic effects. Apoptosis induction observed after TLR4 downregulation in both colorectal cancer cells confirmed previous studies describing the major role of TLR4 in apoptosis resistance and in colon cancer cell survival [33, 35]. In this current study, TLR4 blockade using neutralizing antibody alleviated HM-inhibited ERK phosphorylation, demonstrating that HM acts partially through TLR4 and suggesting the presence of other HM receptors in colorectal cancer cells.

## Conclusions

Our findings have demonstrated that HM inhibits proliferation, induces ROS and apoptosis in human CRC cell lines by activating intrinsic mitochondrial-dependent apoptotic pathway, modulating Bcl2 family protein expression, activating the JNK pathway and inhibiting ERK kinase activity partially through TLR4. These findings indicate that HM might act as a potential anticancer agent against CRC and support the notion that this agent may be effective for the treatment of colorectal adenocarcinoma and mCRC. Nevertheless, further studies are warranted to investigate its potential anti-proliferative and anti-cancerous activities in vivo.

## Data Availability

All data generated or analyzed during this study are included in the manuscript.
